# Interaction of lipoprotein lipase polymorphisms with body mass index and birth weight to modulate lipid profiles in children and adolescents: the CASPIAN-III Study: Interação de polimorfismos da lipoproteína lipase com índice de massa corporal e peso ao nascer para modular o perfil lipídico em crianças e adolescentes: o estudo CASPIAN-III

**DOI:** 10.1590/1516-3180.2015.00792608

**Published:** 2016-01-19

**Authors:** Gholamreza Askari, Motahar Heidari-Beni, Marjan Mansourian, Mohammad Esmaeil-Motlagh, Roya Kelishadi

**Affiliations:** I PhD. Assistant Professor, Discipline of Nutrition, Food Security Research Center, Department of Community Nutrition, School of Nutrition and Food Sciences, Isfahan University of Medical Sciences, Isfahan, Iran.; II Doctoral Student, Discipline of Nutrition, Food Security Research Center, Department of Community Nutrition, School of Nutrition and Food Sciences, Isfahan University of Medical Sciences, Isfahan, Iran.; III PhD. Assistant Professor, Discipline of Biostatistics, Department of Biostatistics and Epidemiology, School of Health, Isfahan University of Medical Sciences, Isfahan, Iran.; IV MD. Professor of Pediatrics, Department of Pediatrics, Ahvaz Jundishapur University of Medical Sciences, Ahvaz, Iran.; V MD. Professor of Pediatrics, Child Growth and Development Research Center, Research Institute for Primordial Prevention of Non-communicable Disease, Isfahan University of Medical Sciences, Isfahan, Iran.

**Keywords:** Lipase lipoproteica, Índice de massa corporal, Peso ao nascer, Polimorfismo genético, Interação gene-ambiente

## Abstract

**CONTEXT AND OBJECTIVE:**

Interactions between body mass index (BMI), birth weight and risk parameters may contribute to diseases rather than the individual effects of each factor. However this hypothesis needs to be confirmed. This study aimed to determine to what extent variants of lipoprotein lipase (LPL) might interact with birth weight or body weight in determining the lipid profile concentrations in children and adolescents.

**DESIGN AND SETTING:**

Substudy of the third survey of a national surveillance system (CASPIAN-III Study) in Iran.

**METHODS:**

Whole blood samples (kept frozen at -70 °C) were randomly selected from 750 students aged 10-18 years. Real-time polymerase chain reaction (PCR) and high-resolution melt analysis were performed to assess S447X (rs328), HindIII (rs320) and D9N (rs1801177) polymorphisms.

**RESULTS:**

The *AG/GG* genotype in D9N polymorphism was associated with higher LDL-C (low-density lipoprotein cholesterol) and lower HDL-C (high-density lipoprotein cholesterol) concentration. Significant interactions were found for D9N polymorphism and birth weight in association with plasma HDL-C concentration, and also for D9N polymorphism and BMI in association with plasma triglyceride (TG) and HDL-C levels. HindIII polymorphism had significant association with birth weight for HDL-C concentration, and with BMI for TG and HDL-C levels. Significant interactions were found for S447X polymorphism and BMI in association with plasma TG and HDL-C concentrations.

**CONCLUSION:**

We found significant interactive effects from LPL polymorphisms and birth weight on HDL-C concentration, and also effects from LPL polymorphisms and BMI on TG and HDL-C concentrations.

## RESUMO

**CONTEXTO E OBJETIVO:**

Interações entre índice de massa corporal (IMC), peso ao nascer e parâmetros de risco podem contribuir para doenças, em vez de efeitos individuais de cada fator. No entanto, essa hipótese precisa de confirmação. Este estudo visou determinar o quanto variantes de lipoproteína lipase (LPL) podem interagir com peso de nascimento ou peso corporal na determinação das concentrações do perfil lipídico em crianças e adolescentes.

**DESENHO E LOCAL:**

Sub-estudo da terceira pesquisa de sistema nacional de vigilância (Estudo CASPIAN-III) no Irã.

**MÉTODOS:**

Foram selecionadas aleatoriamente amostras de sangue total (mantidas congeladas a -70 °C) de 750 estudantes com idades entre 10-18 anos. Reação de polimerase em cadeia (PCR) em tempo real e análise de fusão de alta resolução foram realizados para avaliar polimorfismo de S447X (rs328), HindIII (rs320) e D9N (rs1801177).

**RESULTADOS:**

Genótipo AG/GG em polimorfismo D9N foi associado com concentração maior de LDL-C (colesterol do tipo lipoproteína de baixa densidade) e menor de HDL-C (colesterol do tipo lipoproteína de alta densidade). Interações significativas foram encontradas para polimorfismo D9N e peso ao nascer em associação com concentração plasmática de HDL-C, bem como para polimorfismo D9N e IMC em associação com níveis plasmáticos de triglicérides (TG) e HDL-C. Polimorfismo HindIII teve associação significativa com peso de nascimento para concentração de HDL-C, e com IMC para níveis de TG e HDL-C. Interações significativas foram encontradas para polimorfismo S447X e IMC em associação com concentrações plasmáticas de TG e HDL-C.

**CONCLUSÃO:**

Encontramos efeitos interativos significativos de polimorfismo LPL e peso de nascimento sobre concentração de HDL-C, bem como efeitos de polimorfismos LPL e IMC sobre concentrações de TG e HDL-C.

## INTRODUCTION

Adverse levels of serum lipoprotein cholesterols among children and adolescents are important risk factors for coronary artery and early stages of atherosclerosis. Serum lipid and lipoprotein levels in childhood are generally good predictors of their concentrations in young adulthood.^1^ Elevated triglycerides (TG) and depressed HDL-C are the most common abnormalities of lipids and lipoproteins associated with obesity. This situation has been named atherogenic dyslipidemia.^2^

Some important genetic disorders increase the susceptibility to chronic diseases. Nonetheless, body weight, lifestyle habits and environmental factors, including the intrauterine environment, are important in determining the disease process.^3^^,^^4^

Several enzymes are involved in the metabolism of serum lipids. Lipoprotein lipase (LPL) has an important role in metabolism and transporting of lipids. It plays a major role in hydrolysis of chylomicrons and very low-density lipoproteins. The LPL gene has 10 exons and is located on chromosome 8p22. Recently, a number of more common single-nucleotide polymorphisms (SNPs) in the LPL gene have been described. These are related to lipid concentration, e.g. S447X (*C*--›*G* nucleotide 1595), HindIII (+495T > *G*) and D9N (*G*--›*A* nucleotide 280). It has been found that D9N has small deleterious effects on lipid profiles, whereas S447X has small beneficial effects on serum lipids in adults.^5^

Among perinatal characteristics, unfavorable birth weight and further growth trajectory are the most important factors relating to metabolic abnormalities.^6^ The association between birth weight and the components of metabolic syndrome and other risk factors of cardiovascular disease (CVD) during childhood and adolescence remains controversial. Some studies have reported strong associations between low or high birth weight and the risk of CVD, while others have not found any association.^7^^,^^8^ It has been suggested that some genes involved in metabolic processes may have different effects on people with different birth weights. Investigation on whether an adverse intrauterine environment and birth weight might alter the expression of genes relating to the lipid profile is clinically relevant.^9^^,^^10^ Ruiz et al. hypothesized that some genes (APOE, APOC3 and PPARγ2 genes) might interact with birth weight to determine the blood lipid profile later in life. Their results suggested that the intrauterine environment would interact with the genetic background to determine plasma lipid profile levels in later life.^10^

Genetic factors are considered to be important determinants of plasma lipoprotein levels in adults. However, the role of genetics in determining plasma lipoproteins in children and adolescents is less clear. The increasing prevalence of dyslipidemia in children may be due to complex interactions between genetic and environmental factors. Studies have reported interactions between the LPL gene and lifestyle, in association with lipid profile concentrations in adults.^11^^,^^12^ However, to the best of our knowledge, interactions with factors like birth weight have not been examined.

Some LPL genotypes have been found to have significant associations with changes to TG and HDL-C levels in obese subjects. These findings support the need for further studies to investigate the role of these polymorphisms in obesity.^13^ The interaction between body mass index (BMI) and the LPL genotype may explain the deleterious role of obesity on lipid profile levels.^14^

Interactions between BMI, birth weight and risk parameters may contribute towards diseases, rather than the individual effects of each factor. However, this hypothesis needs to be confirmed. The link between BMI or birth weight and plasma lipid levels has been documented. Nonetheless, the interactions of single nucleotide polymorphisms (SNPs) and BMI or birth weight on plasma lipid levels are limited, particularly in children and adolescents.

## OBJECTIVE

This study aimed to determine to what extent variants of LPL genes might interact with birth weight or body weight in determining the blood lipid profile concentration in children and adolescents.

## METHODS

### Study population

This study was conducted as a substudy of the “school-based nationwide health survey”, which was the third survey of the school-based surveillance system named the Childhood and Adolescence Surveillance and PreventIon of Adult Non-communicable disease (CASPIAN-III) Study.

The survey was conducted among 5570 students aged 10-18 years who were recruited by means of multistage random cluster sampling from urban and rural areas of 27 provincial counties in Iran. Students who had any chronic disease or who were taking medications were not included in this study. Complete data were obtained from 5528 students (2726 girls, 69.37% urban, mean age 14.7 ± 2.4 years) and were reported. Details of the data collection and sampling were published previously.^15^ For the current study, we randomly selected 750 whole blood samples that had been kept frozen at -70 °C.

This survey was approved by ethics committees and other relevant national regulatory organizations. Written informed consent was obtained from parents and oral assent from the children and adolescents involved.

### Physical examination and biochemical measurements

Weight and height were measured in accordance with standard protocols using calibrated instruments. Body mass index (BMI) was calculated as the weight (kg) divided by the height squared (m^2^). According to the World Health Organization (WHO) definition, normal weight was defined as a BMI Z-score between 1 and -2, wasting as a BMI Z-score between -2 and -3, risk of overweight as a BMI Z-score between 1 and 2, overweight as a BMI Z-score between 2 and 3, and obesity as a BMI Z-score of more than 3.^16^ Birth weight was categorized as low birth weight (less than 2,500 g), normal (2,500-4,000 g) or high birth weight (more than 4,000 g).

To assess blood lipid levels, students were invited to go to the healthcare center nearest to their school. Fasting venous blood samples was taken. High-density lipoprotein cholesterol (HDL-C) and triglycerides (TG) were measured using auto-analyzers. HDL-C was measured after precipitation of non-HDL-C with dextran sulfate-magnesium chloride. The low-density lipoprotein cholesterol (LDL-C) levels in serum samples with TG < 400 mg/dl were calculated in accordance with the Friedewald equation. Total cholesterol (TC) was measured by means of an autoanalyzer.

The biochemical analyses were performed in the central provincial laboratory in each county. The methods used in these laboratories were in accordance with those of the National Reference laboratory, which is a WHO-collaborating center in Tehran.^15^

### DNA extraction

DNA was extracted from peripheral blood using the QIAamp DNA blood mini-kit (Qiagen, Germany), in accordance with the manufacturer’s protocol. Real-time polymerase chain reaction (PCR) and high-resolution melt (HRM) analyses were performed in the Corbett rotor-gene 6000 device (Corbett Research Pty Ltd, Sydney, Australia). Primers were designed using Beacon Designer 7.91 with the aim of flanking the genomic regions (Premier Biosoft International, USA) and were synthesized by TIB MOLBIOL (Germany).

Amplicons from all the genes were generated under the following conditions, using the Type-it HRM kit (Qiagen, Germany): one cycle at 95 °C for 15 minutes; 40 cycles at 95 °C for 15 seconds, 60 °C for 15 seconds and 72 °C for 15 seconds; and one cycle of 95 °C for 1 second, 72 °C for 90 seconds and a melt from 70 °C to 95 °C rising at 0.1 °C per second. The amplification mixture had a total volume of 25 µl and included 12.5 µl of HRM PCR master mix, 1.75 µl of 10 µM primer mix, 2 µl of genomic DNA as template and 8.25 µl of RNase-free water. For each genotype reaction, we included sequence-proven major and minor allele homozygote and heterozygote controls. The HRM analysis was performed using instrument software, which allowed clustering of the samples into groups based on difference plots that were obtained by analyzing the differences in melting curve shape between known controls and samples. The primer sequence used for LPL S447X rs328 was F: GCAGAAAGGAAAGGCACCTG, R: CAGGATGCCCAGTCAGCT; for LPL HindIII rs320, it was F: TCCAAGATAATCTCAACCT, R: TAACAATAA- CAGCACACTATA; and for LPL D9N rs1801177, it was F: TCCAAGATAATCTCAACCT, R: GGAATGAGG- TGGCAAGTG.

### Statistical analysis

The data were described by calculating the frequencies (percentages), means and standard deviation (SD). The differences between general characteristics based on LPL polymorphism levels were tested by means of the independent t test for quantitative variables and the chi-square test for qualitative variables. Separate analysis of covariance (ANCOVA) was done using each lipid profile as the dependent variable. Interactions between LPL gene variants and birth weight or BMI were assessed by using a cross-product term between genotypes and birth weight or BMI, on serum lipid levels that adjusted for age, sex, and physical activity. Statistical significance was evaluated through ANCOVA by using a custom model. Chi-square tests were used to assess Hardy-Weinberg expectations. The statistical analyses were performed using the SPSS statistical software package (version 20.0, SPSS Inc., Chicago, Illinois, USA). P-values of less than 0.05 were considered statistically significant.

## RESULTS

The characteristics of the study participants according to genotypes are presented in Table 1. The P-values for Hardy-Weinberg expectations were 0.419 for D9N polymorphism, 0.105 for HindIII polymorphism and 0.06 for S447X polymorphism. The genotypic distribution of the three SNPs was in Hardy-Weinberg equilibrium (P > 0.05 for all). Overall, subjects with the variant allele (*AG* or *GG* genotype) and those with the *AA* genotype in D9N polymorphism did not differ significantly according to sex, age, fasting blood pressure (FBS), systolic blood pressure (SBP), diastolic blood pressure (DBP), TC, TG or physical activity. LDL-C concentration and BMI were significantly higher among those with the *AG* or *GG* genotype than among those with the *AA* genotype. HDL-C concentration was significantly lower among those with the *AG* or *GG* genotype than among those with the *AA* genotype.

**Table 1.**
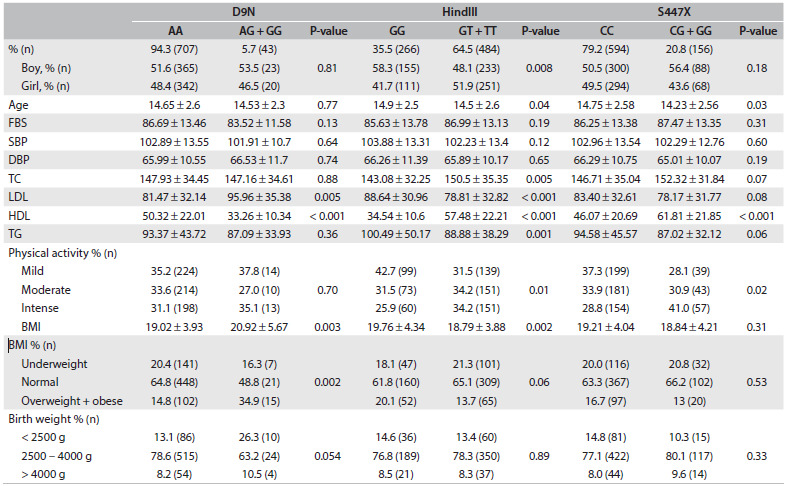
Characteristics of the study population across the lipoprotein lipase gene polymorphisms: the CASPIAN-III study

FBS = fasting blood pressure; SBP = systolic blood pressure; DBP = diastolic blood pressure; TC = total cholesterol; LDL = low-density lipoprotein; HDL = high-density lipoprotein; TG = triglyceride; BMI = body mass index.

Compared with carriers of *GG* in HindIII polymorphism, those with *GT* or *TT* were slightly younger and had higher HDL-C and TC and lower TG and LDL-C concentrations and BMI (P < 0.05). No significant difference existed with regard to sex, FBS, SBP or DBP, between the *GG* and *GT/TT* genotype groups. Physical activity was significantly different between the groups. Those with the *GT/TT* genotype had higher percentages of physical activity in each category (mild, moderate or intense) than those with the *GG* genotype.

There were no significant differences in relating to age, FBS, DBP, SBP, TC, LDL-C, TG or BMI between the *CC* genotype and the *CG/GG* genotype in S447X polymorphism. Compared with carriers of *CC* in S447X polymorphism, those with the *CG/GG* genotype were slightly younger (14.23 ± 2.56 versus 14.75 ± 2.58; P = 0.03) and had higher HDL-C concentration (61.81 ± 21.85 versus 46.07 ± 20.69; P < 0.001). Physical activity was significantly different between the groups. Those with the *CC* genotype had higher percentages of physical activity in each category (mild, moderate or intense) than those with the *CG/GG* genotype.

The interaction effects between LPL genetic variants and birth weight on serum lipid levels after adjustment for age, sex, physical activity and BMI are presented in Table 2. In stratified analyses, the association between the LPL polymorphisms and plasma lipid profile concentrations varied according to the birth weight.

**Table 2.**
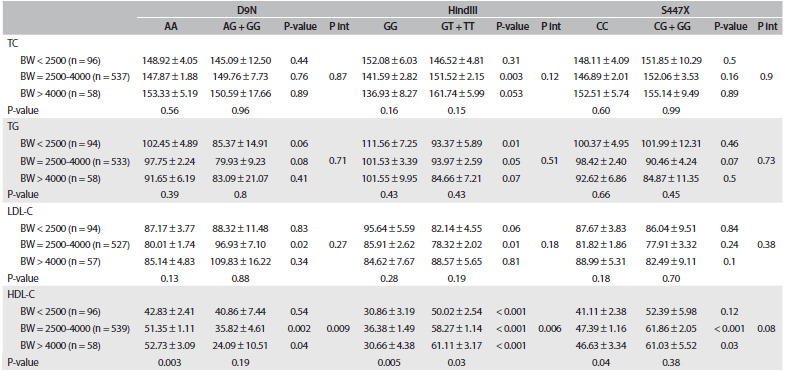
Interactions between lipoprotein lipase gene variants and birth weight, in relation to serum lipid levels in Iranian children and adolescents: the CASPIAN-III study

P_int_ = P interaction; TC = total cholesterol; BW = birth weight; TG = triglyceride; LDL-C = low-density lipoprotein; HDL-C = high-density lipoprotein. Data are mean ± Standard Error (SE). Model adjusted for age, sex, physical activity and BMI.

Significantly higher LDL-C and lower HDL-C concentration in relation to the *G* allele was confined to individuals with normal birth weight, in D9N polymorphism. For instance, among subjects with normal birth weight, plasma HDL-C concentration was significantly lower in those with the *AG/GG* genotype than in those with the *AA* genotype (35.82 ± 4.61 and 51.35 ± 1.11 mg/dl, respectively; P = 0.002). We observed a strong interaction between D9N polymorphism and birth weight in relation to HDL-C concentration.

Among those who had normal birth weight, the *T* allele in HindIII polymorphism was associated with significantly higher TC concentration (*GG* compared with *GT/TT*: 141.59 ± 2.82 and 151.52 ± 2.15 mg/dl, respectively; P = 0.003). This association was marginally significant among those with high birth weight (P = 0.053). Subjects with low birth weight and the *GT/TT* genotype had lower TG level (P = 0.01). This association was marginally significant among those with normal birth weight (P = 0.05). Only among normal birth weight subjects was the T allele associated with significantly depressed LDL-C concentration (P = 0.01). We observed a strong interaction between HindIII polymorphism and birth weight in relation to HDL-C concentration. Subjects with low, normal and high birth weight and the *GT/TT* genotype had higher HDL-C concentration than those with the *GG* genotype (P < 0.001).

Combinations of carrying the *G* allele in S447X polymorphism with normal or high birth weight were significantly associated with higher HDL-C concentration. No significant interaction was documented between birth weight and S447X polymorphism in relation to lipid profile concentrations.

The interaction effects between LPL genetic variants and BMI on serum lipid level, after adjustment for age, sex, physical activity and birth weight, are presented in Table 3. We observed interaction between D9N polymorphism and BMI in relation to TG and HDL-C concentrations (P < 0.001 and P = 0.04, respectively). Underweight and normal-weight individuals with the *AG/GG* genotype had lower HDL-C level than those with the *AA* genotype (P = 0.01).

**Table 3.**
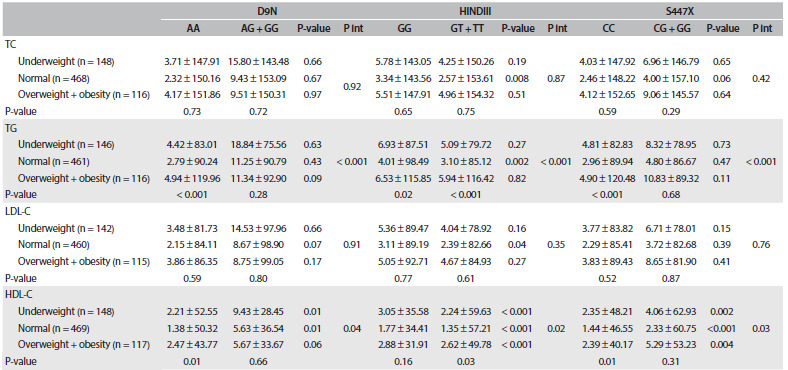
Interactions between lipoprotein lipase gene variants and body mass index, in relation to serum lipid levels in Iranian children and adolescents: the CASPIAN-III study

P_int_ = P interaction; TC = total cholesterol; TG = triglyceride, LDL-C = low-density lipoprotein; HDL-C = high-density lipoprotein. Data are mean ± Standard Error (SE). Model adjusted for age, sex, physical activity and birth weight.

In joint analysis, combinations of carrying the *T* allele (*GT/TT* genotype) with normal weight were significantly associated with higher TC and HDL-C levels, and also with lower TG and LDL-C concentrations, than those of subjects with the *GG* genotype in HindIII polymorphism. Among underweight and overweight/obese subjects, carrying the *T* allele was associated with higher HDL-C concentration than that of subjects with the *GG* genotype (P < 0.001). Moreover, interaction between HindIII polymorphism and BMI was found for TG and HDL-C concentrations (P < 0.001 and P = 0.02, respectively).

Underweight, normal weight and overweight/obesity in subjects with the *G* allele were associated with elevated HDL-C concentrations in S447X polymorphism. Interaction between S447X polymorphism and BMI existed in relation to TG and HDL-C concentrations (P < 0.001 and P = 0.03, respectively).

Linear regression models after adjustment for confounders showed that D9N polymorphism was significantly positively related to LDL-C in the normal birth weight group (â ± standard error (SE) = 18.51 ± 6.57; P-value = 0.005), and significantly negatively related to HDL-C in the normal and > 4000 g birth weight groups (â ± SE = -17.68 ± 4.52; P-value = < 0.001; and â ± SE = -26.53 ± 5.02; P-value = 0.03, respectively). However, D9N polymorphism was not significantly associated with total cholesterol and TG in any of the birth weight groups. HindIII polymorphism was positively related to HDL-C and negatively related to TG, in all the birth weight groups. In addition, HindIII had a beneficial effect on LDL-C (â ± SE = -9.98 ± 2.85; P-value = 0.001) in the normal birth weight group. Thus, HindIII polymorphism may improve lipid profiles. S44X polymorphism was significantly positively associated with HDL-C alone, in all the birth weight groups (birth weight < 2500 g: â ± SE = 11.36 ± 4.34; P-value = 0.003; birth weight 2500-4000 g: â ± SE =15.31 ± 2.19; P-value = 0.001; and birth weight > 4000 g: â ± SE = 16.89 ± 3.02; P-value = 0.01). In different BMI categories, a significant adverse association was found between D9N polymorphism and HDL-C (underweight: â ± SE = -19.49 ± 8.44; P-value = 0.02; normal: â ± SE = -17.44 ± 4.88; P-value = < 0.001; and overweight + obese: â ± SE = -11.46 ± 5.22; P-value = 0.02). A significant association was found for HindIII and S447X polymorphisms in relation to elevated HDL-C, in all BMI categories. In addition, HindIII polymorphism had a beneficial effect on other lipid profiles in the normal BMI category (these data were not shown).

## DISCUSSION

We showed that the *AG/GG* genotype in D9N polymorphism is associated with higher LDL-C and lower HDL-C concentrations. However, these associations were observed only among individuals who had normal birth weight, and among underweight and normal-weight subjects according to BMI. Significant interactions were found for D9N polymorphism and birth weight in association with plasma HDL-C concentrations, and also for D9N polymorphism and BMI in association with plasma TG and HDL-C levels. Our data suggest that the effect of LPL polymorphisms on the lipid profile is modulated by either birth weight or BMI.

One case-control study in Australia showed that D9N polymorphism did not significantly influence HDL-C or TG.^17^ Other studies showed that D9N polymorphism had an adverse effect on serum lipids, and increased the risk of CVD.^18^^,^^19^ It is possible that D9N polymorphism leads to deficiency of LPL secretion.^17^

The importance of the gene/environment interaction has been discussed in relating to human disease processes and disease prevention. Regulation of lipid profile concentrations is a complicated and poorly understood process that may depend on interactions of both environmental and genetic factors. Researchers have hypothesized that the interactions between some genetic polymorphisms and environmental factors may be related to serum lipid levels.^20^^,^^21^ Several studies have investigated the influence of weight or birth weight on plasma lipid levels.^22^ LPL polymorphisms also affect lipid concentrations.^23^ However, the interaction between BMI or birth weight and the polymorphisms of LPL, on serum lipid levels, is not well known, particularly among children and adolescents. It is noteworthy to mention that in the current study, HindIII polymorphism had a significant association with birth weight, in relation to HDL-C concentration, and with BMI in relation to TG and HDL-C levels. The *GT/TT* genotype in HindIII polymorphism is associated with higher TC and HDL-C and lower TG and LDL-C concentrations.

HindIII polymorphism is a thymine to guanine base transition at position +495 in intron 8 and is one of the most common polymorphisms in the LPL gene.^24^ The association between HindIII polymorphism and TG is more controversial. While some studies have shown significant associations between HindIII polymorphism and hypertriglyceridemia,^25^^,^^26^ these findings have not been confirmed by others.^27^^,^^28^ However, most studies have proposed that this polymorphism is a modulator of plasma lipid levels.^29^^,^^30^ The mechanism of its effect on plasma lipid levels remains to be determined.^24^

In this study, plasma HDL-C levels were significantly higher in individuals with the *G* allele (*CG* or *GG* genotype) than in those with the *CC* genotype in S447X polymorphism. Significant interactions were found for S447X polymorphism and BMI in association with plasma TG and HDL-C concentrations.

Nettleton et al.^31^ showed that white and African-American adults with the *CG/GG* genotype had higher HDL-C and lower TG concentrations than their counterparts. This profile is generally associated with reduced risk of CVD. Likewise, a case-control study in India confirmed that in CVD patients, Ser447X polymorphism was associated with decreased TG levels and that this effect was possibly due to increased LPL activity.^30^ A meta-analysis on previous studies among adults showed that S447X polymorphism decreased TG and increased HDL-C, and therefore decreased the CVD risk.^32^

The key role of LPL is in postprandial lipid metabolism. However, it is also involved in metabolism of fasting lipid. S447X polymorphism affects postprandial lipemia, and this may be a mechanism for its effects on TG and HDL-C concentrations.^31^

However, the mechanism for the effects of this polymorphism on serum lipid metabolism remains obscure. One study suggested that S447X polymorphism might alter LPL translation,^33^ but its mechanism is unclear.^32^

A previous study speculated that the gene encoding LPL played an important role in dyslipidemia in an Asian population.^13^ LPL has a central role in lipid metabolism. There is a clear relationship between LPL activity and lipid concentrations. Insulin is a major regulator of LPL activity. Since insulin levels and activity are related to body weight, it can be hypothesized that LPL activity is affected by obesity. It has been shown that LPL polymorphisms affect plasma lipid concentration to a greater or lesser degree in individuals with different BMI levels.^13^^,^^14^

To the best of our knowledge, no previous study has reported on interactions between LPL polymorphisms and birth weight or between LPL polymorphisms and BMI interactions, in relation to serum lipid concentrations in children and adolescents. It is plausible that interactions between birth weight or BMI and the LPL polymorphisms in association with serum lipid concentrations might occur through their effects on LPL activity.

Studies have shown that there are low TG concentrations in overweight and obese individuals who carry the *T* allele, which in turn might be due to increased LPL activity. Furthermore, overweight and obese individuals with the *GG* genotype have been found to have higher serum TG levels than their non-obese counterparts.^11^ A cross-sectional study showed that, among obese subjects, carrying the S447X allele alone was associated with lower TG concentrations. Thus, TG levels were modified significantly by interactions between LPL polymorphism and BMI in adults.^34^ We also observed significant interactions between LPL polymorphisms and BMI in association with lipid profile in children and adolescents.

On the other hand, another study that investigated interactions between obesity and the S447X polymorphism showed that subjects with S447X polymorphism and normal BMI had significantly lower TG concentration. However, no significant difference was documented for those with excess weight.^35^ In another study, the HindIII polymorphism was strongly correlated with hypertriglyceridemia in obese subjects.^36^

The responsiveness of LPL to glucose or insulin stimuli is delayed in the adipose tissue of obese subjects. Therefore, it is supposed that obesity might lead to a condition that may increase genetic susceptibility to hyperlipidemia and diabetes.^37^

It has been found that birth weight might affect cardiovascular events and the components of metabolic syndrome.^38^^,^^39^ Some researchers have proposed that this relationship is genetically mediated.^40^ Among adult men, higher birth weight has been correlated with decreased TC levels.^41^ Several studies have reported a U-shaped association between birth weight and cardiovascular risk factors in children and adolescents.^42^^,^^43^ Numerous cohort and experimental studies on animals have confirmed that fetal programming of adult diseases exists, and have suggested that undernutrition during fetal life and low birth weight can lead to future disease.^44^^,^^45^ Reduced fetal growth may have adverse consequences on liver growth. Poor liver growth may cause disorders of blood lipid metabolism.^10^

However, the underlying molecular and genetic mechanisms of the association of birth weight with disease in later life are not clearly understood.^44^ We observed significant interactions between LPL polymorphisms and BMI in association with some lipid profiles in children and adolescents. No previous data from children are available for comparison.

Interactions between BMI and LPL polymorphisms or between birth weight and LPL polymorphisms may explain how subjects with a certain genotype fail to maintain homeostasis and ideal levels of plasma lipids only after the environmental challenge of increasing obesity. Our results showed that the differences in the lipid profile levels of underweight, normal weight and overweight/obese subjects or between birth weight categories might be partly because of different interactions between some SNPs and BMI or birth weight in the population studied.

According to the linear regression model, our findings suggest that there is a beneficial association between HindIII or S447X polymorphism and lipid profile, particularly with regard to HDL-C levels, in all categories of birth weight or BMI. We can conclude that these two polymorphisms may improve lipid profiles in children and adolescents. However, D9N had undesirable effect on lipid profiles.

Study limitations and strengths: The main limitation of this study is the cross-sectional nature of the associations. Other possible study limitations include the small sample size in some subgroups, particularly in the groups of individuals with birth weight > 4000 g and with the *AG/GG* genotype for D9N. The strengths of this study are its novelty in the pediatric age group, and its inclusion of a relatively large number of population-based samples.

## CONCLUSION

We observed that children and adolescents with the HINDIII and S447X alleles had better serum lipid profiles than those of non-carriers. We found significant interactive effects between LPL polymorphism and birth weight, in relation to HDL-C concentrations, and also significant effects between LPL polymorphism and BMI in relation to TG and HDL-C concentrations. The clinical significance of these associations needs to be confirmed in future longitudinal studies.
